# Continued Weight Loss and Sarcopenia Predict Poor Outcomes in Locally Advanced Pancreatic Cancer Treated with Chemoradiation

**DOI:** 10.3390/cancers11050709

**Published:** 2019-05-23

**Authors:** Patrick Naumann, Jonathan Eberlein, Benjamin Farnia, Thilo Hackert, Jürgen Debus, Stephanie E. Combs

**Affiliations:** 1 Department of Radiation Oncology, University Hospital Heidelberg, Im Neuenheimer Feld 400, 69120 Heidelberg, Germany; jonathan.eberlein@med.uni-heidelberg.de (J.E.); juergen.debus@med.uni-heidelberg.de (J.D.); 2 Clinical Cooperation Unit Radiation Oncology, dkfz Heidelberg, Im Neuenheimer Feld 280, 69120 Heidelberg, Germany; 3 Department of Radiation Oncology, University of Miami, 1475 NW 12th Avenue, Suite 1500, Miami, FL 33136, USA; benjamin.farnia@jhsmiami.org; 4 Department of General, Visceral and Transplantation Surgery, University Hospital Heidelberg, Im Neuenheimer Feld 110, 69120 Heidelberg, Germany; thilo.hackert@med.uni-heidelberg.de; 5 Deutsches Konsortium für Translationale Krebsforschung (DKTK), Core Center Heidelberg, 69120 Heidelberg, Germany; 6 Department of Radiation Oncology, Technical University Munich (TUM), Ismaninger Straße 22, 81675 München, Germany; stephanie.combs@tum.de; 7 Institute of innovative Radiotherapy (iRT), Department of Radiation Sciences (DRS), Helmholtz Zentrum München (HMGU), Ingolstädter Landstraße 1, 85764 Oberschleißheim, Germany; 8 Deutsches Konsortium für Translationale Krebsforschung (DKTK), Partner Site Munich, 81675 München, Germany

**Keywords:** locally advanced pancreatic cancer, chemoradiation, weight loss, muscle wasting, body composition, skeletal muscle index, sarcopenia, cachexia

## Abstract

*Background*: Surgical resection offers the best chance of survival in patients with pancreatic cancer, but those with locally advanced disease (LAPC) are usually not surgical candidates. This cohort often receives either neoadjuvant chemotherapy or chemoradiation (CRT), but unintended weight loss coupled with muscle wasting (sarcopenia) can often be observed. Here, we report on the predictive value of changes in weight and muscle mass in 147 consecutive patients with LAPC treated with neoadjuvant CRT. *Methods*: Clinicopathologic data were obtained via a retrospective chart review. The abdominal skeletal muscle area (SMA) at the third lumbar vertebral body was determined via computer tomographic (CT) scans as a surrogate for the muscle mass and skeletal muscle index (SMI) calculated. Uni- and multi-variable statistical tests were performed to assess for impact on survival. *Results*: Weight loss (14.5 vs. 20.3 months; *p* = 0.04) and loss of muscle mass (15.1 vs. 22.2 months; *p* = 0.007) were associated with poor outcomes. The highest survival was observed in patients who had neither cachectic weight loss nor sarcopenia (27 months), with improved survival seen in those who ultimately received a resection (23 vs. 10 months; *p* < 0.001). Cox regression revealed that either continued weight loss or continued muscle wasting (SMA reduction) was predictive of poor outcomes, whereas a sarcopenic SMI was not. *Conclusions*: Loss of weight and lean muscle in patients with LAPC is prognostic when persistent. Therefore, both should be assessed longitudinally and considered before surgery.

## 1. Introduction

In recent years, survival rates have dramatically increased in the field of oncology following the evolution of novel treatment approaches, including advances in surgical resection, radiotherapy, and systemic treatments, particularly with multidrug chemotherapy and immunotherapy [1]. Nevertheless, patients with pancreatic adenocarcinoma continue to have a dismal prognosis with largely unchanged 5-year overall survival rates of 9%, irrespective of stage [1,2]. Presently, the best chance of survival among this cohort is a complete surgical resection in the absence of metastatic disease [3,4]. However, due to a lack of early symptomatology, most patients often present with locally advanced disease (LAPC), preventing a surgical approach.

LAPC is defined as solid tumor contact of epigastric vessels that precludes surgical resection. In this cohort, neoadjuvant multidrug chemotherapy and/or chemoradiation (CRT) are often employed to downstage patients, with subsequent resection rates of 40–60% observed [5,6,7,8]. Indeed, this approach has been endorsed by the National Comprehensive Cancer Network (NCCN) for all patients who are not deemed surgical candidates, have no metastasis and have a reasonable performance status.

Patients with LAPC typically present with a history of weight loss, often caused by a variety of symptoms: loss of appetite, epigastric pain, malabsorption, reduced gastrointestinal motility and/or cholestasis caused by tumor growth. We have previously found that weight loss and a reduction in subcutaneous fat mass predicts for reduced overall survival [8]. Weight loss and muscle wasting, also known as sarcopenia, are considered typical signs of cancer cachexia [9,10]. In fact, according to international consensus, cancer cachexia is defined as either a relative weight loss of greater than 5% or sarcopenia in combination with weight loss of at least 2% [9]. The term sarcopenia is commonly used to describe the phenomenon of muscle wasting in frail patients or patients suffering from cancer and is defined as a depletion in muscle mass resulting in a skeletal muscle index (SMI) below gender specific cut-off values that might even occur in obese patients and is then termed sarcopenic obesity [11].

Indeed, a diagnosis of sarcopenia has been found to be a negative predictor for outcome following surgical resection in patients with pancreatic cancer [12,13,14]. Similarly, survival rates were even lower in pancreatic cancer patients with sarcopenia who have metastatic disease [15].

While weight loss and muscle wasting both play an integral role in overall outcome, the treatment approach may impact these variables in different ways. In patients with LAPC who receive neoadjuvant chemotherapy, a mean reduction in body composition and skeletal muscle area was found to be less than 5 and 0.5%, respectively, underscoring a propensity to conserve muscle mass [16]. Contrastingly, neoadjuvant CRT used for patients with resectable and borderline resectable disease was found to lead to reduced skeletal muscle area [17].

Several efforts have been made in the nutritional sciences to quantify body composition variables in an attempt to mitigate this treatment and disease-related burden. For instance, a newly introduced variable, the psoas index, defined as the area of both psoas muscles normalized to the third lumbar vertebral body, was found to correlate with improved outcomes in patients with resectable disease [18]. Beyond a reduction in muscle area, an additional fatty muscle degeneration, termed myosteatosis, has also been associated with worse outcomes [19]. One recently published series of patients showed that patients treated preoperatively with either chemotherapy or CRT who had a gain in muscle mass were more likely to undergo resection [20]. However, there is a paucity of data on the impact of sarcopenia in patients with LAPC, especially among those treated with CRT, which is the subject of the present study.

## 2. Results

Our retrospective cohort was comprised of 147 consecutive patients treated with neoadjuvant CRT for unresectable LAPC. Patient characteristics are shown in Table 1.

The treatment course consisted of conventional photon irradiation with a median dose of 54 Gy (range, 45–55 Gy) combined with once weekly gemcitabine (300 mg/m^2^ body surface area (BSA)). Treatment was completed within an average of 38 days. Following treatment completion, patients received an additional cycle of gemcitabine (1000 mg/m^2^ BSA) until first follow-up. The average time from planning CT to first follow-up CT was 2.6 months (range, 1.8–4.0 months) and this reflects the study period for body composition analysis.

### 2.1. Weight Loss and Changes in Skeletal Muscle Area

**Figure 1:**
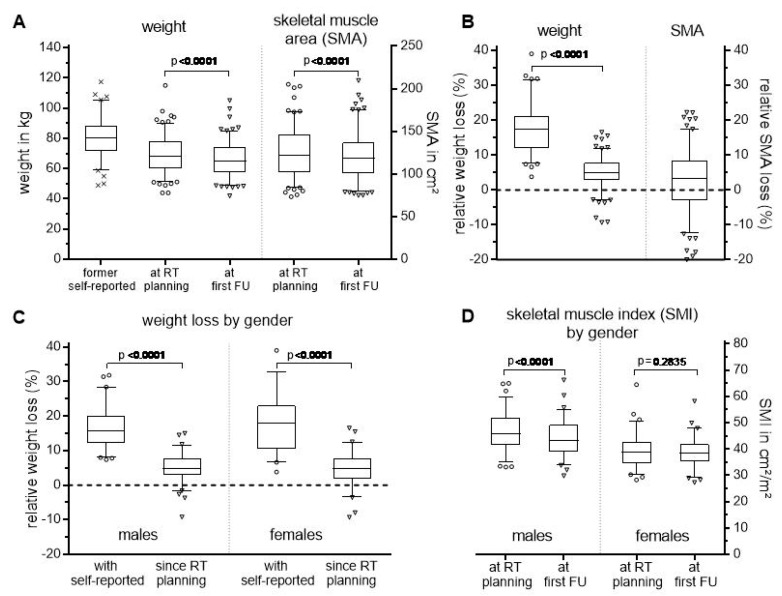
Absolute (**A**) and relative (**B**) changes in weight and skeletal muscle area (SMA) measured prior to treatment initiation, and at the time of planning CT for radiation (RT) and first follow-up (FU). (**C**) Changes in relative weight loss and (**D**) skeletal muscle index (SMI) according to gender.

Prior to treatment initiation, the mean self-reported weight was 80.4 kg (range, 49.0–117.3 kg). At the time of planning CT for radiation treatment, the average weight was reduced to 69.7 kg and continued to decrease by an average of 3.7 kg for the entire cohort until first follow-up (*p* < 0.0001, Figure 1A), corresponding to a mean reduction in body mass index (BMI) from 27.5 to 24.1 and finally to 22.8 kg/m^2^ (range, 17.2–46.7 kg/m^2^). This weight loss reflects a relative decrease by 17.3% (max. 39%) and 6.1% (max. 16.5%) when compared to the self-reported value and that measured in our department at the time of planning CT, respectively (Figure 1B).

To further characterize the patient’s nutritional status, we determined the skeletal muscle area (SMA) and calculated the corresponding skeletal muscle index (SMI) via normalization to the patient’s height. At follow-up, the SMA was absolutely and relatively reduced by a mean of 4.2 cm^2^ (max. 35 cm^2^) and 2.7% (max. 22%) (*p* < 0.0001, Figure 1A,B). The reduction was gender dependent, with no statistically significant difference seen in females (Figure 1D). In contrast, the mean weight loss was similar for both sexes (Figure 1C).

**Figure 2:**
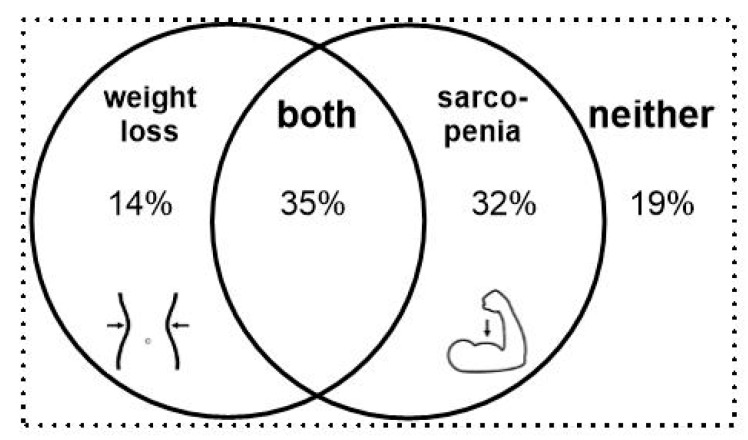
Distribution and combination of weight loss and sarcopenia observed between planning CT and first follow-up.

Utilizing its definition, tumor cachexia occurred in 49% of our cohort during the treatment course (Figure 2).

Rates of cachexia increase to 85% when based on initial, self-reported body weight. Sarcopenia occurred in 67% of our patients. The combination of cachectic weight loss and sarcopenia was identified in 35%, but jumped to 60% if based on initial, self-reported body weight. There was a sub-group of patients (19%), comprised mostly of women (64% vs. just 36% men), who showed neither cachectic weight loss nor sarcopenia.

Table 2 summarizes the clinical parameters of patients with and without cachectic weight loss or sarcopenia, including the analysis for statistical significance.

Clinical parameters were largely balanced among groups. However, patients with weight loss >5% during CRT had a lower initial CA 19.9, higher BMI at CT simulation and were less likely to have surgical exploration, with no difference seen in resection rates. Patients with sarcopenia were more often male, with a lower performance status (less ECOG 0) and lower BMI and were more likely to utilize high caloric drinks for nutritional support. There were no differences in resection rates on sarcopenia status. Muscle density and intramuscular fat area tended to be higher and lower, respectively. Nutritional support was used in nearly half of the cohort (39%) to treat weight loss, with 62% receiving parenteral nutrition and the remaining 38% utilizing high-caloric drinks, without significant improvement. Oral nutritional support was utilized in 18% vs. 15% and parenteral nutritional support was utilized in 30% vs. 21% for patients with or without cachexia (weight loss > 5% and sarcopenic SMI’s), respectively.

### 2.2. Treatment-Related Toxicity

**Figure 3:**
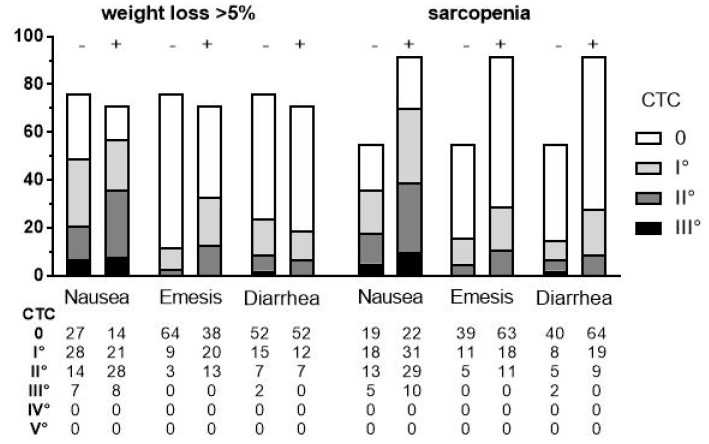
Distribution of National Comprehensive Cancer Network (NCCN) Common Toxicity Criteria (CTC) grades for nausea, emesis and diarrhea during chemoradiation (CRT) grouped according to the presence or absence of cachectic weight loss and sarcopenia, respectively.

To assess whether CRT induced nausea, emesis or diarrhea that may have contributed to weight loss and muscle waste, we retrospectively analyzed patient charts for these symptoms, which were graded according NCCN Common Toxicity Criteria (CTC) version 5.0 (Figure 3).

Patients with weight loss or sarcopenia had higher rates of grade 2 nausea, whereas those with sarcopenia experienced mild to moderate diarrhea more often. Moreover, emesis of any grade was more frequent in patients with weight loss as well as with sarcopenia.

### 2.3. Survival Analysis

Five patients (3%) who died following surgical complications (pulmonary artery embolism × 2, sepsis × 2, and fungal pneumonia) were excluded from survival analysis.

**Figure 4:**
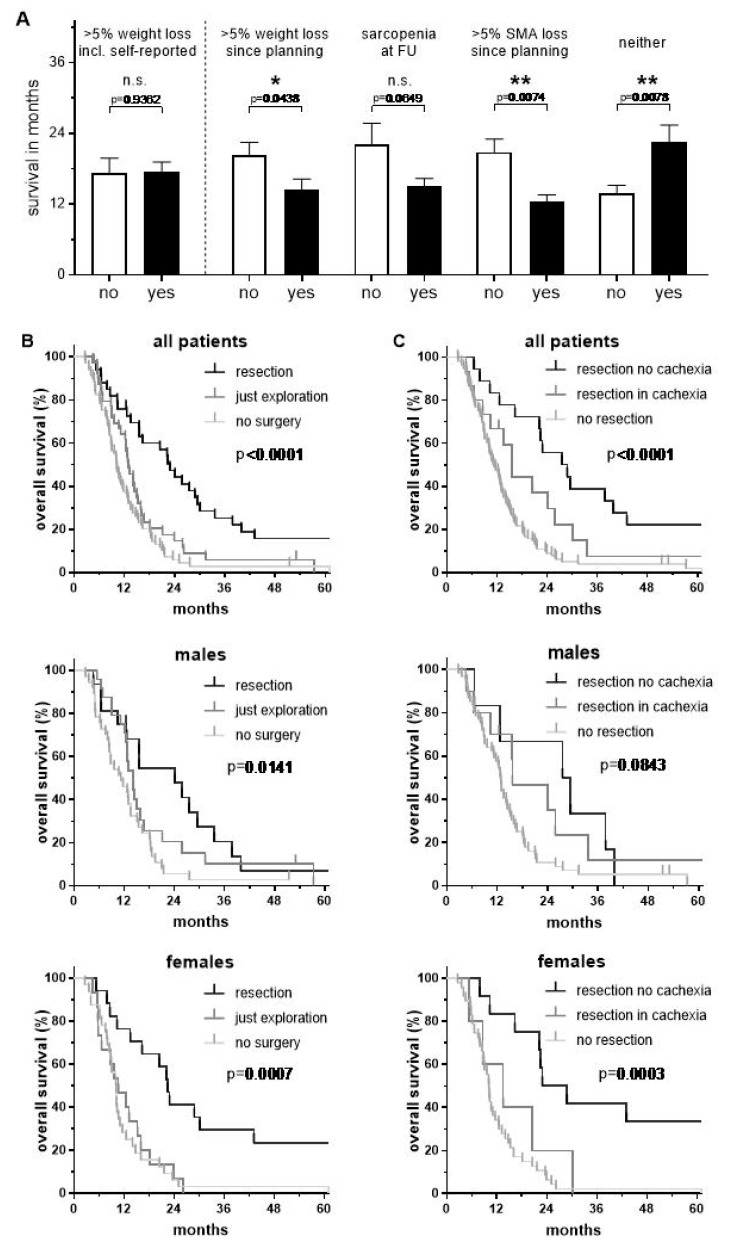
(**A**) Average overall survival according to status of cachectic weight loss and/or sarcopenia. (**B**) Kaplan–Meier survival curves grouped according to the extent of surgical resection and (**C**) in combination with or without cachexia as well as by gender.

Average overall survival was lower among patients with a cachectic weight loss, a sarcopenic SMI or a SMA loss > 5% from CRT until first follow-up. All these variables reached statistical significance except sarcopenia, where just a trend (*p* = 0.0649) was observed due to the higher standard error of the mean. Patients having neither weight loss nor sarcopenia at first follow-up had the highest mean survival (27.1 vs. 15.1 months, *p* = 0.0027). Importantly, self-reported weight loss prior to CRT had no impact on survival (Figure 4A).

After CRT, 36 patients (25%) had a successful resection with a median survival of 22.9 months and 5-year overall survival rate of 16%. In contrast, patients who underwent surgical exploration and were still deemed unresectable (*n* = 41, 29%) had a median survival of 13.0 months. Similarly, those patients who were still unresectable at first follow-up or who refused to have surgery (*n* = 65, 46%) had a median survival of 10.2 months and 5-year survival rate of 3% (Figure 4B). If the final resection was performed, the majority of patients needed reconstructive vessel surgery (*n* = 24, 67%). Patients underwent arterial resections primarily of the hepatic artery, venous resections primarily of the portal vein or a combination of both with subsequent reconstruction in 28% (*n* = 10), 14% (*n* = 5) and 25% (*n* = 9) of patients, respectively. The involved vessels of the remaining 12 patients (33%) were conserved since only fibrotic tissue was left after CRT that could be surgical removed.

Survival was highest among patients who had no cachexia, with a median survival of 27.5 months and a 5-year survival rate of 23.5%, and was comparably lower among those with cachexia, where the median survival was 15.6 months (Figure 4C). Interestingly, survival curves after resection and in particular in the absence of cachexia were more disparate for women than men (Figure 4B,C).

**Figure 5:**
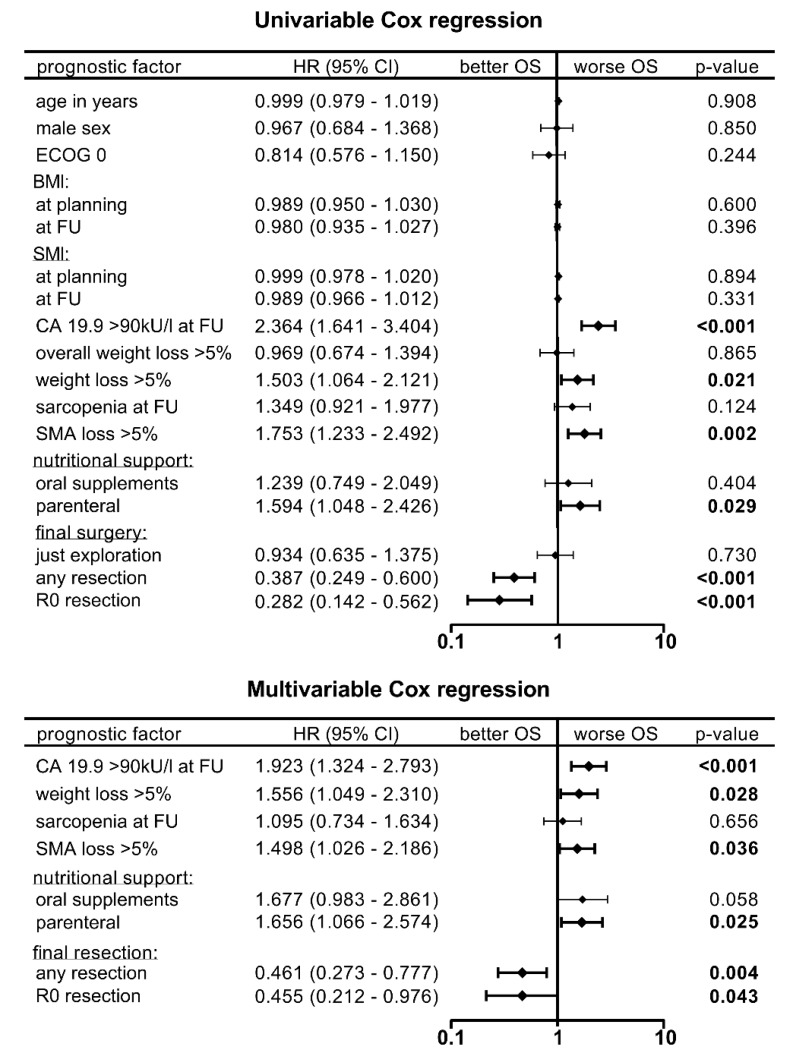
Uni- and multivariable Cox regression analysis, with hazard ratios (HR) and a 95% confidence interval (CI). Factors with *p*-values < 0.15 in univariable cox regression were selected for multivariable analysis.

To evaluate the hazard ratios (HR) of different factors for survival, we performed uni- and multivariable Cox regression analysis (Figure 5).

In the univariable analysis, the following variables were identified as predictors for poor overall survival: CA 19.9 value > 90 kU/L, >5% loss of weight or SMA, and nutritional support by parenteral infusion. In contrast, sarcopenic SMI was not significant. Final surgical resection was one of the most important prognostic factors, regardless of R-status (*p* < 0.001, HR 0.4 and 0.3, respectively). In multivariable analysis, the aforementioned variables remained significant, independent predictors, with sarcopenic SMI continuing to lack significance.

## 3. Discussion

The present study is a retrospective review of a homogeneous cohort diagnosed with LAPC, who had an initial unresectable disease without evidence of metastatic burden and received neoadjuvant CRT. The majority of patients had persistent weight loss combined with sarcopenia as determined by measurements of skeletal muscle area in CT scans at two distinct time points: before CRT and at first follow-up for re-evaluation of resectability. Patients with continued weight loss and sarcopenia reported higher rates of nausea and vomiting during CRT. Having a weight loss > 5%, sarcopenic SMI or a reduction in the SMA > 5% at first follow-up or a combination of the above significantly reduced mean survival. Importantly, patient-reported weight loss prior to treatment initiation did not impact on survival. Survival rates were highest among patients who underwent subsequent resection and had neither weight loss nor sarcopenia. However, Cox proportional hazards regression revealed that sarcopenia as defined by SMI below gender-specific cut-off values is not a predictor of survival, whereas persistent loss of muscle mass or weight were negatively associated with survival, such as CA 19.9 at follow-up and parenteral nutritional support. Furthermore, any subsequent resection, regardless of extent, was identified as a positive predictor for survival whereas just an exploration was not.

Weight loss is a common problem in patients with pancreatic cancer and can negatively affect outcome as previously reported [8]. In our series, weight loss of 5% or more that corresponds to tumor cachexia was observed in 49% during CRT and in 85% when patient-reported weight loss prior to treatment initiation was included. This is in line with other reports among patients with newly diagnosed disease or when receiving neoadjuvant treatment, where rates of 62 to 72%, respectively, were identified [17,21]. Similarly, the majority of patients in our cohort (68%) had sarcopenia, comparable to published ranges of 55–65% for mostly resectable pancreatic cancer [13,14,16,17,19]. Comparable with previously published reports, sarcopenia and weight loss were negatively associated with survival in our cohort. However, our cohort showed a more pronounced reduction in muscle mass (2.7%) compared to previously published data (0.5%) for patients who underwent neoadjuvant treatment [16]. However, it should be highlighted that our study is novel in reporting outcomes in patients with locally advanced disease only, as the overwhelming body of evidence has focused on patients with earlier stage disease that is more often amenable to surgical resection. Another recent series of patients with pancreatic cancer treated with mostly chemotherapy and CRT in some showed a reduction in adipose tissue but not muscle mass [20], underscoring differences depending on disease stage and treatment modality. In addition to muscle wasting, a fatty degeneration of lean muscle termed myosteatosis was reported to impact on outcomes [19]. However, our cohort did not show any changes in muscle density determined in Hounsfield units of the SMA. In fact, intramuscular fat area even tended to be slightly reduced at follow-up.

Although patients with sarcopenia at first follow-up had reduced survival, we found that having reduced SMIs did not follow suit in Cox regression models. In contrast, a decrease of at least 5% of the SMA during CRT was a predictor of survival. This might be explained when considering that some patients experienced a beneficial increase in their muscle area during CRT, but SMI remained below criteria and thus still met sarcopenic definition. Moreover, the SMI cut-offs to be defined as having sarcopenia are highly dependent on ethnicity, given different criteria proposed for Western and Eastern populations as recently assessed [22]. In contrast to our data, in a recent series of patients with borderline resectable disease and LAPC, no loss of muscle mass was observed during neoadjuvant treatment and a subgroup of ultimately resected patients actually had an increase in skeletal muscle area by 5.9% [20]. Therefore, we believe that sarcopenia is best judged longitudinally by assessing changes in the SMA with at least two CT images.

Interestingly, statistical analysis revealed differences in survival according to gender, with more disparate survival curves for females who finally underwent a resection compared to males, especially when no cachexia was present. Nevertheless, interpretation of this observation is difficult given that patient numbers in these gender sub-subgroups were relatively low.

In our cohort, nutritional support was utilized by 39% with either nutritional infusions or high-caloric drinks. Nevertheless, loss of muscle mass and weight was persistent for many patients, highlighting a discrepancy to the benefits of postoperative nutritional support and counseling seen in several prospective phase I/II trials [23]. For instance, early postoperative enteral nutrition was shown to be superior to parenteral infusions [24]. In line with this, the prescription of parenteral nutrition in our cohort was significantly associated with poorer overall survival (*p* = 0.025). Whether this is a direct association or reflects a poorer performance status cannot be clearly differentiated. Moreover, patient compliance to nutritional prescriptions as well as real-time caloric intake documentation were not available. Given that rates of nutritional support were higher in patients with cachexia, these patients might have a more rapid metabolism.

Importantly, among patients without cachexia who underwent a resection, the mean survival rates of 27.5 months were comparable to those seen in large clinical trials, including European Study Group for Pancreatic Cancer (ESPAC)-3 and ESPAC-4 which examined the role of adjuvant chemotherapy among patients who underwent resection in the first place [25,26]. The fact that weight loss and rates of sarcopenia seem to be similarly distributed among those with resectable and unresectable disease and survival is equal when resection was performed supports the idea that—if no metastasis is evident—every unresectable patient should be treated in the neoadjuvant setting in an attempt to achieve later resectability. Nevertheless, before a decision is made to recommend surgical intervention, biomarkers such as CA 19.9 [27], in conjunction with the patient’s performance status, weight loss and changes in muscle area, should be examined. Interestingly, initial CA 19.9 values of our cohort were significant lower in the subgroup of patients with cachectic weight loss (621 ± 949 vs. 1994 ± 4579; *p* = 0.020) although one might expect the opposite. However, we believe that this is subject to selection bias since patients with weight loss and high CA 19.9 values were presumably in a worse condition and therefore more likely transferred to palliative chemotherapy instead of neoadjuvant CRT.

Despite the weakness of our retrospective study design, we see a strength in the homogeneous population of patients with LAPC who had measurements of weight and muscle mass at two distinct time points, enabling a longitudinal analysis of body composition and associated changes over time to more reliably assess for any underlying loss. Although single-slice CT measurements are widely used, with established results correlating with total body muscle mass, another limitation of our study is that we did not collect data on muscle strength, which typically declines in patients with sarcopenia and cachexia and could strengthen the findings presented. Additional research is needed to validate the data presented.

## 4. Materials and Methods

### 4.1. Patient Selection and Treatment Approach

Utilizing our institutional database, we retrospectively identified 147 consecutive patients with LAPC who were treated with neoadjuvant CRT at our department between 2007 and 2014. Inclusion criteria included having a planning CT simulation as well as CT for first follow-up, and a re-evaluation of resectability (typically 4–6 weeks after completion of irradiation). Histologic confirmation was required for all patients. However, if the biopsy results were not conclusive and a repeat biopsy was not possible or denied by the patient, LAPC status was determined on the basis of interdisciplinary tumor board recommendations, integrating radiologic imaging and CA 19.9 levels characteristic of LAPC. Resectability was assessed according to NCCN criteria. Patients with stage IV disease prior to the initiation of neoadjuvant CRT were excluded. CRT was delivered with photons utilizing conventional fractionation combined with once weekly gemcitabine 300 mg/m^2^ BSA, followed by one cycle of gemcitabine 1000 mg/m^2^ BSA until first follow-up for the reevaluation of resectability. The study was approved by our institutional ethics committee (S-483/2011 and S-063/2019 according new data protection regulations).

### 4.2. CT Analysis, Sarcopenia, and Cachexia

Skeletal muscle area (SMA) was determined at the mid plane of the third lumbar vertebra in both CTs (the first for planning simulation before treatment and the second at first follow-up after treatment) for each patient using the software SliceOmatic^®^ 5.0 (Tomovison^®^, Magog, QC, Canada). As previously reported, this vertebral level is considered the best reference site to assess lean muscle [28] and was recently confirmed to have a good correlation with other established methods, including bioelectrical impedance analysis and dual x-ray absorptiometry [29]. The threshold for muscle tissue was set to −29 to +150 HU as previously reported [30]. The resulting areas were manually checked and corrected by two independent physicians. Final areas comprised the muscles of the abdominal wall, the back and lumbar area, namely the rectus abdominis, external oblique, transversus abdominis, internal oblique, psoas, quadratus lumborum and erector spinae muscles. Due to threshold settings, intramuscular fat was excluded but its area measured separately. The skeletal muscle index (SMI) was calculated for each patient by normalizing the measured SMA to the square of the patient’s height. As suggested previously, sarcopenia was defined when the SMI was below the sex-specific cut-off values of 38.5 and 52.4 cm^2^/m^2^ for females and males, respectively [11,31]. Finally, according to international consensus, cancer cachexia was defined when patients have either a weight loss of greater than 5% or a depletion in muscle mass below these gender specific cut-offs in combination with persistent weight loss of at least 2% [9].

### 4.3. Statistical Analysis

Prism 7.04 software (GraphPad, San Diego, CA, USA) and SPSS Statistics version 24(International Business Machines Corporation, Armonk, New York, NY, USA) were used to perform statistical analysis and graphical plotting of results. Results were considered significant when the *p*-value was less than 0.05. For the continuous variables of weight, SMA and SMI, D’Agostino and Pearson omnibus K2 normality tests suggested a Gaussian distribution and paired *t*-tests were used to compare means of groups for statistical difference. Overall survival was calculated using Kaplan–Meier and log-rank tests. Average overall survival in different groups were compared using nonparametric Kolmogorov–Smirnov tests since D’Agostino and Pearson omnibus K2 normality tests revealed that overall survival rates did not follow a Gaussian distribution. Finally, a Cox regression analysis was performed assuming proportional hazards. A univariable model was first utilized to identify possible prognostic factors for overall survival, with promising variables subsequently selected for a multiple cox regression analysis to test the overall model. Hazard ratios corresponding to the 95% confidence interval were calculated.

## 5. Conclusions

In conclusion, neoadjuvant chemoradiation can enable successful surgical resection with prolonged survival in patients with locally advanced pancreatic cancer, but if cachectic weight loss and muscle depletion is observed, surgery may have less impact on survival. Therefore, an assessment of these variables should be incorporated in determining whether surgical resection should take place. To assess sarcopenia more reliably, muscle mass should be measured at two time points rather than utilizing fixed cut-offs of skeletal muscle index. Given that loss of weight and muscle mass directly impact outcomes among this cohort, prompt and thorough nutritional counseling may be essential in this patient population.

## Figures and Tables

**Table 1:** Demographics, baseline parameters and treatment durations.

Characteristics	Mean (SD)
Age, in years	63.6 (±9.0)
Gender, *n* (%)	
Male	79 (53.7%)
Female	68 (46.3%)
Tumor Site, *n* (%)	
Head	84 (57.2%)
Body	29 (19.7%)
Tail	1 (0.7%)
Multiple	33 (22.4%)
Tumor Stage, *n* (%)	
I	0 (0%)
II	0 (0%)
III	147 (100%)
IV	0 (0%)
ECOG score, *n* (%)	
0	75 (51.0%)
1	60 (40.8%)
2	12 (8.2%)
3	0 (0%)
Prior chemotherapy	
None	126 (85.8%)
Gemcitabine	8 (5.4%)
Gemcitabine + Erlotinib	5 (3.4%)
Gemcitabine + Cisplatin	1 (0.7%)
Capecitabine	1 (0.7%)
FOLFIRINOX	3 (2.0%)
Unknown	3 (2.0%)
Height, in centimeter	170 (±9.6)
Weight, in kg (SD; range)	69.7 (±12.3; 44–115))
BMI, in kg/m² (SD; range)	24.1 (±3.8; 17.2–46.7)
BMI WHO class distribution, *n* (%)	
Underweight (BMI < 18.5)	3 (2.1%)
Normal (18.5 ≤ BMI < 25 kg/m^2^)	89 (60.5%)
Pre-obese (25 ≤ BMI < 30 kg/m^2^)	47 (32.0%)
Obesity (≥30 kg/m^2^)	8 (5.4%)
Median CA 19.9, in kU/L (SD; range)	230.3 (±3272; 0.1–27,031)
Timeline, in days (SD)	
Planning CT to treatment initiation	10.6 (±6)
Treatment duration	38.3 (±4)
Treatment completion to follow-up CT	29.4 (±9)
Planning CT to follow-up CT	78.3 (±11)

ECOG: Eastern Cooperative Oncology Group, FOLFIRINOX: folinic acid, fluorouracil, irinotecan andoxaliplatin, BMI: body mass index, WHO: World Health Organization, CA: Carbohydrate antigen, CT: computer tomogramm.

**Table 2:** Clinical parameters grouped according to cachectic weight loss and sarcopenia.

Parameter	Weight Loss > 5%			Sarcopenia		
	No (*n* = 76)	Yes (*n* = 71)	*p*-Value	No (*n* = 48)	Yes (*n* = 99)	*p*-Value
Age, in years	63.6 ± 9.3	63.4 ± 8.8	0.922	62.9 ± 10.2	63.9 ± 8.5	0.552
Sex, % of men	40 (52.6%)	39 (54.9%)	0.939	14 (29.2%)	65 (65.7%)	<0.001
ECOG 0	41 (53.9%)	34 (47.9%)	0.451	32 (66.7%)	43 (43.4%)	0.004
CA 19.9, in kU/L						
Initial	1994 ± 4579	621 ± 949	0.020	1692 ± 4476	1106 ± 2637	0.359
FU	709 ± 1880	808 ± 1,959	0.778	545 ± 1,204	852 ± 2155	0.412
SMA, in cm/m^2^						
CT Simulation	124.8 ± 32.1	127.2 ± 24.2	0.619	129.7 ± 33.3	124.3 ± 25.6	0.299
FU	123.7 ± 29.5	119.4 ± 23.3	0.332	128.5 ± 30.3	118.2 ± 24.1	0.032
SMA density, in HU						
CT Simulation	38.4 ± 9.1	38.6 ± 10.2	0.935	37.6 ± 8.7	38.9 ± 10.0	0.433
FU	40.2 ± 7.3	40.2 ± 5.8	0.980	40.1 ± 7.0	40.3 ± 6.4	0.836
Intramuscular fat area, in cm/m^2^						
CT Simulation	12.7 ± 8.2	12.6 ± 8.4	0.977	13.7 ± 8.0	12.2 ± 8.4	0.302
FU	12.0 ± 7.7	10.4 ± 6.7	0.178	11.8 ± 7.4	10.9 ± 7.2	0.503
Nutritional support						
None	48 (63.2%)	41 (57.7%)	0.181	41 (85.4%)	51 (51.5%)	0.025
High caloric drinks	14 (18.4%)	9 (12.7%)	0.338	3 (6.3%)	19 (19.2%)	0.046
Parenteral	14 (18.4%)	21 (29.6%)	0.082	7 (14.6%)	26 (26.3%)	0.134
IMRT-technique	31 (40.8%)	19 (26.8%)	0.679	15 (31.3%)	35 (35.4%)	0.375
Final surgery						
Exploration	27 (35.5%)	14 (19.7%)	0.034	12 (25.0%)	27 (27.3%)	0.858
Any resection	16 (21.1%)	20 (28.2%)	0.181	10 (20.8%)	23 (23.2%)	0.822
R0 resection	9 (11.8%)	5 (7.0%)	0.325	5 (10.4%)	9 (9.1%)	0.750
BMI, in kg/m^2^						
CT Simulation	23.2 ± 3.1	25.1 ± 4.3	0.003	25.9 ± 4.6	23.4 ± 3.1	<0.001
FU	22.7 ± 3.0	22.9 ± 3.8	0.710	24.4 ± 3.9	22.1 ± 2.9	<0.001
